# Supplementary material for: END-TO-END, FULLY AUTOMATED MANUFACTURING OF HEMATOPOIETIC STEM AND PROGENITOR CELL THERAPY VIA CCR5-TARGETED INTEGRATION
Source: bioRxiv. 2026 Oct 30:2026.10.28.758978. Preprint. [Version 1] doi: 10.64898/2026.10.28.758978 (PMC13733995; doi:10.64898/2026.10.28.758978)
Supplement: Supplementary file 1 [file media-1.pdf]

SUPPLEMENTAL FIGURES

Figure S1.

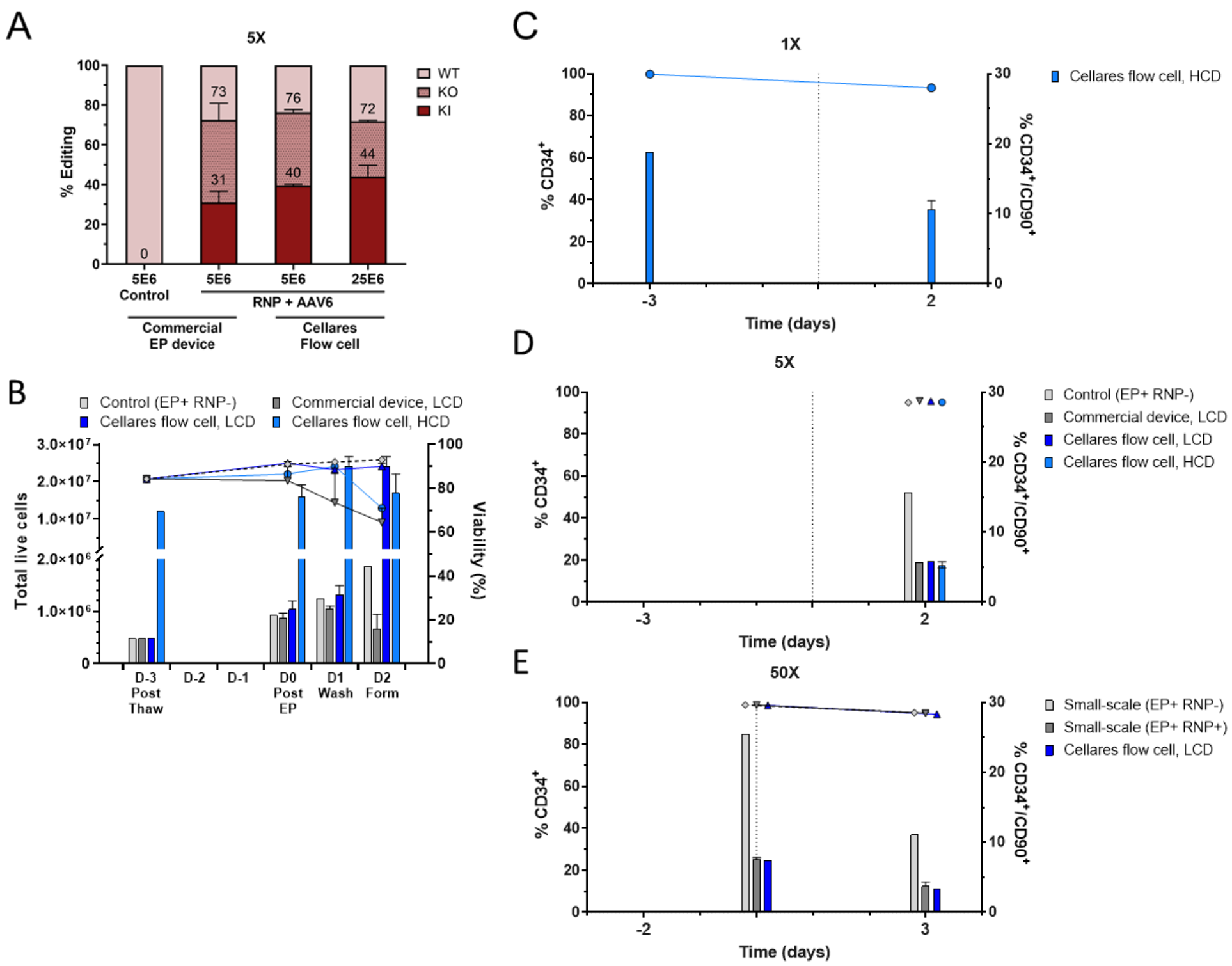

Figure S1. Intermediate EP scale and HSPC purity through scale-up.

(A, B) Editing performance and viability at intermediate EP scale (5x: 500 µL) at HCD ($5x10^7$ cells/mL). (A) KO and KI efficiencies. (B) Total live cells and viability across process time points. (C-E) Frequency of $CD34^+/CD90^+$ (bars, left axis) and $CD34^+$ (lines/points, right axis) populations at different time points. (C) HSPC purity at 1x scale. (D) HSPC purity at 5x scale. (E) HSPC purity at 50x scale. Mean and SD are plotted; n = 1-2 replicates.

Figure S2.

A

B

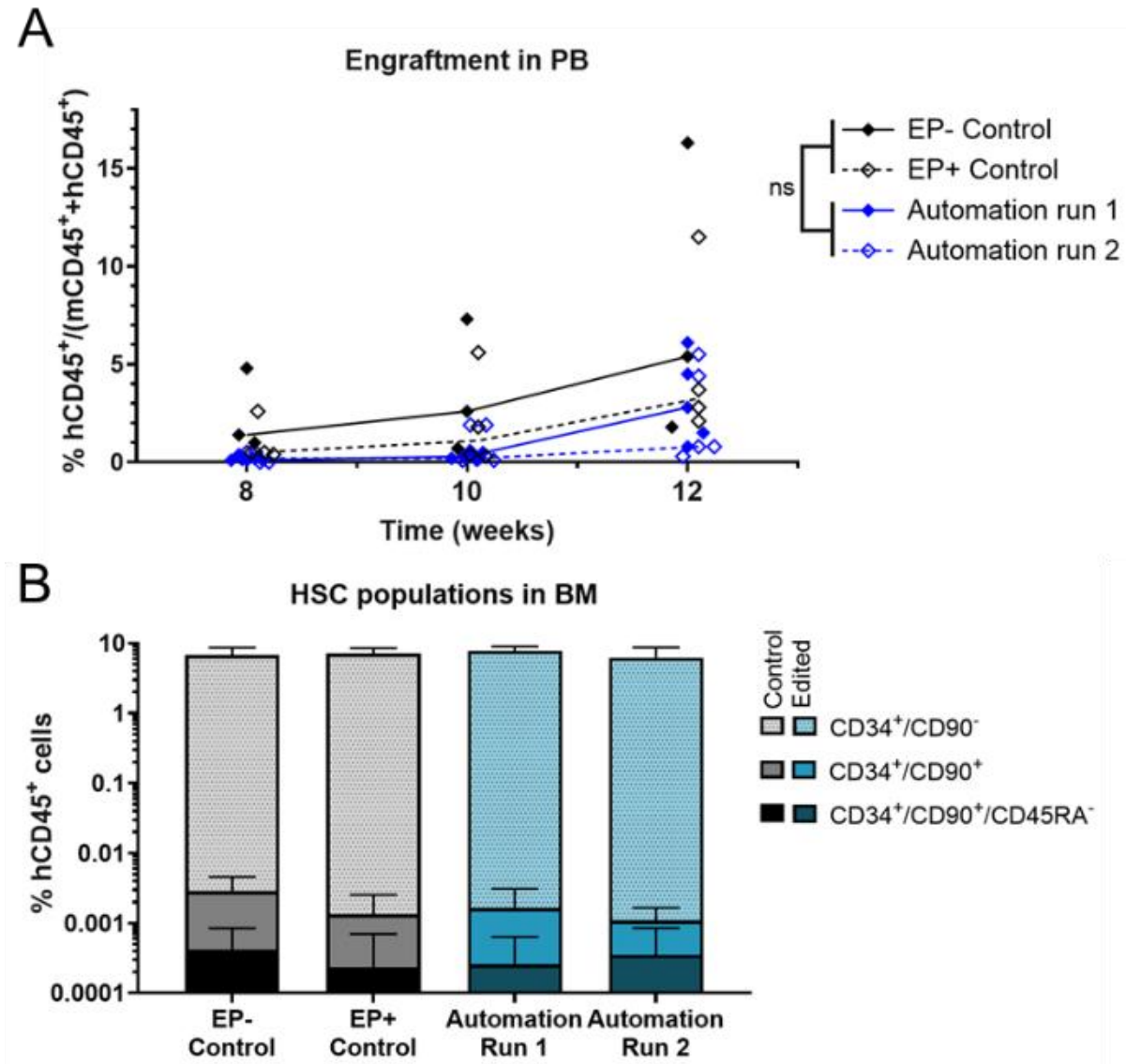
Engraftment in PB
% hCD45+/(mCD45+ +hCD45+)
Time (weeks)
EP- Control
EP+ Control
Automation run 1
Automation run 2
ns
HSC populations in BM
% hCD45+ cells
Control
Edited
CD34+/CD90-
CD34+/CD90+
CD34+/CD90+/CD45RA-
EP- Control
EP+ Control
Automation Run 1
Automation Run 2

Figure S2. Peripheral blood engraftment and HSC populations in bone marrow of NSBGW mice.

(A) Engraftment in PB obtained by retro-orbital bleeds between weeks 8 and 12 as measured by flow cytometry on human and mouse $CD45^+$ cells. (B) Frequency of $CD34^+/CD90^-$, $CD34^+/CD90^+$, and $CD34^+/CD90^+/CD45RA^-$ populations in BM at necropsy. Mean and SD are plotted; n = 3-5 mice; significance calculated by unpaired two-tailed t test at each time point.
